# The mediation effect of individual eating behaviours on the relationship between socioeconomic status and dietary quality in children: the Korean National Health and Nutrition Examination Survey

**DOI:** 10.1007/s00394-016-1184-2

**Published:** 2016-02-26

**Authors:** Hye Ah Lee, Hyesook Park

**Affiliations:** Department of Preventive Medicine, School of Medicine, Ewha Womans University, 1071, Anyangcheon-ro, Yangcheon-ku, Seoul, 158-710 Republic of Korea

**Keywords:** Causal mediation, Children, Dietary quality, Micronutrients

## Abstract

**Purpose:**

Although it has been suggested that socioeconomic status is associated with dietary quality, the possible mediation effects of eating behaviours on dietary quality are unclear. Thus, we investigated the causal chain by which socioeconomic status influences the quality of the diets consumed by children through their eating behaviours using data from the Korean National Health and Nutrition Examination Survey.

**Methods:**

The study focused on persons from 2 to 18 years of age who completed the 24-h dietary recall survey (*n* = 3158). Using causal mediation analysis, we assessed the relationship between socioeconomic status and poor dietary quality in children and examined the mediation effects of eating behaviours. Socioeconomic indicators included household income, parental education, and parental occupation. Dietary quality was defined by the number of key nutrients, protein, calcium, phosphorous, iron, vitamin A, vitamin B1, vitamin B2, niacin, and vitamin C, consumed at insufficient levels.

**Results:**

In the present study, more than half the children did not consume the recommended amounts of vitamin A, vitamin C, iron, and calcium. Eating breakfast had a significant impact on poor dietary quality regardless of socioeconomic indicators. On the other hand, children from lower-middle-income households consumed insufficient amounts of more nutrients than their counterparts regardless of eating behaviours. Through the mediation model, we found that lower-middle household incomes were associated with poor dietary quality, but that dietary quality was significantly mediated by eating breakfast.

**Conclusion:**

We found that poor dietary quality among children in lower-income households was partially explained by their being less likely to eat breakfast, but that eating breakfast did not entirely mediate these effects. Thus, to reduce differences in dietary quality among children, those who are economically vulnerable must be prioritized.

## Background

The trend towards increased consumption of energy dense or convenience food contributes to poor dietary quality [1], and some studies have suggested that this trend has led to widespread health problems such as obesity and chronic diseases. Moreover, in developed countries, insufficient micronutrient intake has become a critical public health issue [2]. Indeed, healthy eating can help children reach their full growth and development potential, especially during critical growth periods [3].

Individual dietary quality may be affected by multidimensional factors, such as preferences, household environment, and socioeconomic status [4, 5]. The complexity of this issue can confuse attempts to determine key factors and causal relationships. One factor potentially related to dietary quality, socioeconomic status, was examined using household income, parental education [6, 7], and occupation [6]. Household income may directly influence food purchases, and the other two factors are linked to knowledge, the understanding of information, and having the time to prepare meals. Other evidence has suggested that habits such as eating breakfast, family meals, and eating out are also associated with dietary quality [8, 9].

The identification of significant modifiable factors is key to improving the average dietary quality of children. Although it has been suggested that socioeconomic status is strongly associated with dietary quality [4, 6], the mediation effects of eating behaviours on the quality of nutrition are unclear. Thus, we investigated the causal chain by which socioeconomic status influences dietary quality through eating behaviours. Using data from the 2010–2011 Korean National Health and Nutrition Examination Survey (KNHANES), we assessed (1) the direct effect of socioeconomic status on dietary quality and (2) the mediation effects of healthy eating behaviours on dietary quality among children and adolescents.

## Methods

### Study subjects

The present study used data from the KNHANES, which is a national survey that has been conducted annually since 2007 to understand the health and nutrition status of the Korean population. The questions pertain to all weeks of the year to avoid issues related to seasonal variation. Data were gathered via a health interview, health examination, and nutrition survey. In the present study, we used the first 2 years of available survey data from the fifth wave of KNHANES (from 2010 to 2012). Survey samples for KNHANES were selected with a multi-stage probability sampling design. To allow inferences to be made from the sample population to the general Korean population, the KNHANES provides individual weight values. The detailed survey method of the KNHANES has already been published [10].

This study was limited to subjects aged 2–18 years. Respondents with missing data on the nutrition survey or with abnormally low or high calorie intake per day (<500 kcal or ≥5000 kcal) were excluded (*n* = 540). Additionally, in accordance with Goldberg’s cut-offs, we excluded subjects who underreported (*n* = 233) [11]. The final study included 3158 children (boys = 1654 and girls = 1504). There were no differences between excluded and included subjects with regard to the distribution of household income from quartile (Q) 1 (low) to Q4 (high) (13.6, 32.1, 30.6, and 23.8 % for included subjects, respectively, vs. 19.8, 33.0, 27.1, and 20.1 % for excluded subjects, respectively; *p* = 0.09). The two groups also did not differ significantly with regard to other socioeconomic indicators or the number of nutrients that were consumed in insufficient quantities (*p* > 0.05). The study protocol was approved by the Institutional Review Board of the Ewha Womans University Hospital.

### Dietary quality

We used data on quantity of nutrition intake derived from the 24-h dietary recall survey based on the weekday before the survey. The dietary survey was conducted by trained dieticians in face-to-face interviews at the participants’ homes. Participants were asked open-ended questions about what they had consumed in the previous 24 h and were able to answer using the help of various measuring aids. Children participated in the interviews with their guardian. Based on previous related studies [12, 13], we selected nine nutrients for examination: protein, calcium, phosphorous, iron, vitamin A, vitamin B1, vitamin B2, niacin, and vitamin C. Individual dietary quality was assessed using the index of nutritional quality (INQ), which was calculated by dividing the nutritional intake per 1000 kcal of total energy intake by the recommended intake (RI) of each nutrient per 1000 kcal. Age- and sex-specific RI were obtained from the 2010 Korean Dietary Reference Intake (KDRI) [14]. An INQ value ≥1.0 indicates that intake of a specific nutrient is sufficient, but an INQ value <1.0 indicates that intake of a specific nutrient is insufficient, even if individuals have reached their total energy requirement. The number of nutrients at insufficient levels was used as a measure of poor dietary quality; because we focused on nine nutrients, poor dietary quality scores ranged from 0 to 9, and higher values reflect poorer dietary quality.

### Socioeconomic indicators

We used data on quartiles of adjusted household income, parental education, and occupation as socioeconomic indicators. Due to differences in consumption-related expenditures according to family size, we used quartiles of adjusted household income, which are calculated as total household income divided by the square root of family size. The first two quartiles were merged into lower-middle household income, and the others were merged into upper-middle household income; these were then defined as “low” and “high” household income, respectively. Parental education level was classified into two levels (graduated high school; some college or higher). Parental occupation was defined as economic inactivity, manual, or non-manual. A negative response to a question about current economic activity was defined as economic inactivity. Manual workers included skilled agricultural, forestry, and fishery workers; craftspeople and related tradespeople; equipment, machine operating, and assembling workers; and unskilled labourers. Non-manual workers included managers, professionals, technicians, and associated professionals, clerks, and service and sales workers.

### Individual eating behaviours

Our study examined eating breakfast, eating with family members, and frequency of eating out. Eating breakfast was defined as having eaten breakfast on both of the 2 days before the survey. Data on eating with family members were collected via the following question: Did you usually eat breakfast/lunch/dinner with your family (more than one person in your family) during the last year? Family meal was defined as at least one affirmative answer in response to questions about breakfast, lunch, and dinner.

The following question addressed eating out: “On average, how often did you eat out during the last year?” The response options were as follows: “twice or more times per day”, “once per day”, “5–6 times per week”, “3–4 times per week”, “1–2 times per week”, “1–3 times per month”, and “less than once per month”. Based on the distribution of responses, respondents were divided into two groups according to the frequency with which they ate out: once or more per day and other (twice or more per day: 9.18 %; once per day: 16.18 %; 5–6 times per week: 69.26 %; and other: 5.38 %).

### Statistical analysis

All statistical analyses were planned in the context of the initial design of the survey. The basic characteristics of study subjects are described as weighted percentages and weighted means. The mediation analysis provides a better understanding of the causal chain by which an independent variable (X) influences a dependent variable (Y) through a mediator (M). Consistent with its conceptual definition [15], this involves sequential testing of the following: (1) the effect of independent variable (X) on dependent variable (Y); (2) the effect of independent variable (X) on mediator (M), (3) the effect of mediator (M) on dependent variable (Y) controlling for independent variable (X), and (4) the effect of independent variable (X) on dependent variable (Y) controlling for mediator (M). When the mediator exerts a significant effect, the direct effect of the independent variable is attenuated, and the statistical significance of this effect can be tested using extant statistics software. We examined statistical significance after controlling relevant covariates. In our study, we assessed the direct and indirect effects of socioeconomic status (X) on poor dietary quality (Y); the indirect effect was mediated by eating behaviours (M). Furthermore, we identified the socioeconomic factors or eating behaviours with a statistically significant contribution to this relationship. Thus, after creating combined variables for socioeconomic status (household income and parental educational level, and occupation) and eating behaviours (eating breakfast, family meals, and eating out), we separately assessed the direct effect of socioeconomic status and the mediation effect of eating behaviours on poor dietary quality in children using the “mediation” package in R statistical software. We adjusted for children’s sex, age, region (urban/rural), total energy intake, and survey year; covariates were selected based on previous studies. All statistical analyses were conducted using the “survey” and “mediation” packages of the R program, version 3.1.1 for Windows.

## Results

The basic characteristics of the study population are presented in Table 1. The average age of subjects was 10.6 years; 53.46 % of subjects were boys, and 46.54 % were girls. The vast majority (83.2 %) were urban dwellers; 39.83 % of mothers and 50.27 % of fathers had some college or higher education. However, mothers were more than twice as likely as fathers to be economically inactive (55.26 vs. 25.49 %). Regarding those with paying jobs, 12.95 % of women and 30.58 % of men had manual occupations, and 31.79 % of women and 43.93 % of men had non-manual occupations. Additionally, 78.14 % children responded that they had eaten breakfast on both of the 2 days before the survey, and 25.38 % had eaten out at least once a day. On average, 3.7 nutrients were not consumed in the amounts suggested by the 2010 KDRI.

**Table 1:** Basic characteristics of study subjects (*N* = 3158)

	Weighted mean (S.E) or weighted %
Age (years)	10.57 (0.14)
Sex	
Boys	53.46 %
Girls	46.54 %
Town	
Urban	83.20 %
Rural	16.80 %
Survey year	
2010	49.47 %
2011	50.53 %
Adjusted household income	
Q1 (low)	13.56 %
Q2	32.11 %
Q3	30.58 %
Q4 (high)	23.75 %
Paternal education	
Graduated from high school	49.73 %
Some college or higher	50.27 %
Maternal education	
Graduated from high school	60.17 %
Some college or higher	39.83 %
Paternal occupation	
Non-manual	43.93 %
Manual	30.58 %
Economic inactivity	25.49 %
Maternal occupation	
Non-manual	31.79 %
Manual	12.95 %
Economic inactivity	55.26 %
Eating meals with family members	
No	13.15 %
Yes	86.85 %
Eating breakfast with family members	
No	33.18 %
Yes	66.82 %
Eating dinner with family members	
No	22.80 %
Yes	77.20 %
Frequency of eating meals with family members per day	
0	13.16 %
1	29.11 %
2	53.99 %
3	3.74 %
Eating breakfast	
No	21.86 %
Yes	78.14 %
Eating out	
≥Once a day	25.38 %
<Once a day	74.62 %
The number of insufficient nutrients	3.68 (0.04)
Total energy (kcal)	1995.57 (19.25)

Weighted percentages estimated in consideration of survey sampling design

**Fig. 1:**
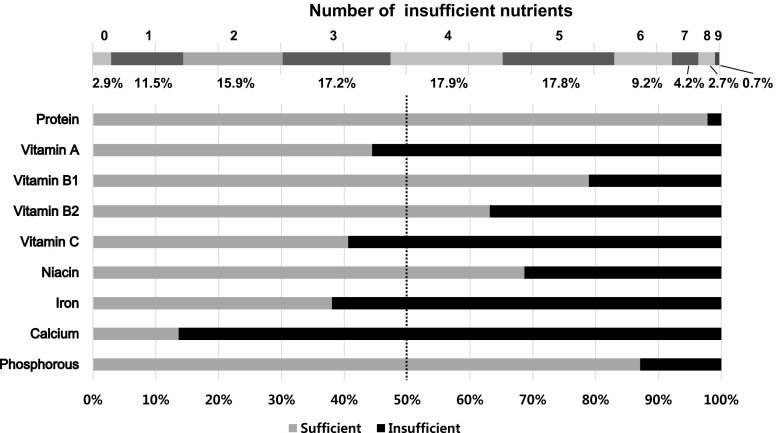
Weighted percentages of insufficient nutrients in Korean children. Nutrient consumption that did not reach the age- and sex-specific recommended intake levels suggested by the 2010 Korean Dietary Reference Intake was defined as insufficient. Weighted percentages estimated in consideration of survey sampling design

The proportions of subjects who had consumed insufficient amounts of the nine nutrients are presented in Fig. 1. More than half the children did not reach recommended intake levels for vitamin A, vitamin C, iron, and calcium. Of the nine nutrients, calcium was the most commonly under-consumed nutrient (86.36 % insufficient intake), followed by iron (61.96 % insufficient intake). About 7.5 % of children did not meet the recommended intake levels for seven or more nutrients.

Table 2 presents the associations between socioeconomic status and eating behaviours. Children with low household income were less likely to eat breakfast and eat out than children with high household income even after adjusting for covariates. However, maternal educational level and occupation were not associated with eating behaviours. Paternal education level and occupation also showed no association in this regard (data not shown).

**Table 2:** Association between healthy eating behaviours and socioeconomic status

Factors	Outcome	Coefficient (S.E)	*p* value
Low household income (ref. high)	Eating breakfast	−0.310 (0.133)	0.02
	Family meal	−0.071 (0.181)	0.69
	Unusual eating out	0.357 (0.143)	0.01
Mother with low education level (ref. some college or higher)	Eating breakfast	−0.035 (0.149)	0.81
	Family meal	−0.034 (0.181)	0.85
	Unusual eating out	−0.039 (0.115)	0.74
Mother engaged in manual work (ref. non-manual and economic inactivity)	Eating breakfast	−0.029 (0.142)	0.84
	Family meal	−0.006 (0.185)	0.74
	Unusual eating out	−0.023 (0.118)	0.85

Coefficients and standard errors obtained from a probit regression model using a generalized linear model function after adjusting for sex, age, region (urban/rural), total energy intake, and survey year. Socioeconomic status indicators and healthy eating behaviours relied on dichotomous data

*S.E* standard error

**Fig. 2:**
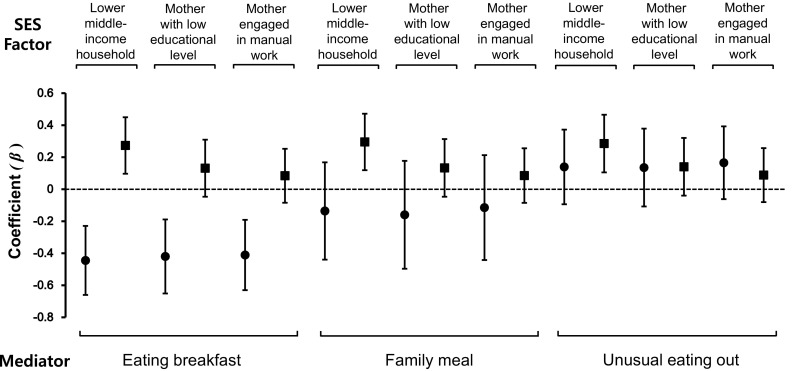
Estimated coefficients for the effects of socioeconomic status and eating behaviours on children’s dietary quality. For assessing poor dietary quality in children, the model included socioeconomic status (household income, maternal educational level, and maternal job, individually) and eating behaviours (eating breakfast, family meal, and eating out individually) as explanatory variables with adjustment for sex, age, total energy intake, region (urban/rural), and survey year. Socioeconomic status and eating behaviours included household income (low vs. high), maternal education level (graduated from high school vs. some college or higher), and occupation (manual vs. other), eating breakfast (yes vs. no), family meal (yes vs. no), eating out (<once a day vs. ≥once a day). The latter indicates the reference group

Figure 2 shows one model of the effects of socioeconomic status and eating behaviours on dietary quality after adjusting for covariates. Eating breakfast was significantly negatively associated with the number of nutrients consumed at insufficient levels regardless of socioeconomic indicators. On the other hand, children with a low household income consumed insufficient amounts of more nutrients than their counterparts regardless of their eating behaviours. However, other eating behaviours and socioeconomic indicators showed no association with poor dietary quality.

Table 3 presents the results of the mediation analysis. The indirect effect of household income on children’s dietary quality through eating breakfast as well as the direct effect of household income was significant, accounting for 6.9 % of children’s poor dietary quality. In addition, when considering other eating behaviours, the significant impact of household income on children’s poor dietary quality remained, but there were no mediation effects of family meals or eating out.

**Table 3:** Direct and indirect effects on dietary quality

Factors	Mediator	ACME		ADE		Proportion of ACME	
		Estimate	95 % CI	Estimate	95 % CI	Estimate	95 % CI
Low household income	Eating breakfast	0.022	0.001, 0.048	0.275	0.097, 0.449	0.069	0.002, 0.225
	Family meal	0.001	−0.007, 0.010	0.293	0.121, 0.466	0.001	−0.031, 0.037
	Unusual eating out	0.008	−0.006, 0.028	0.287	0.100, 0.467	0.023	−0.019, 0.141
Mother with low educational level	Eating breakfast	0.002	−0.022, 0.027	0.128	−0.061, 0.316	0.014	−0.511, 0.600
	Family meal	0.0002	−0.008, 0.009	0.135	−0.052, 0.317	0.00004	−0.173, 0.139
	Unusual eating out	−0.001	−0.013, 0.007	0.137	−0.048, 0.327	−0.002	−0.196, 0.137
Mother engaged in manual work	Eating breakfast	0.002	−0.019, 0.024	0.081	−0.091, 0.247	0.015	−0.747, 0.987
	Family meal	0.001	−0.008, 0.010	0.084	−0.091, 0.265	0.001	−0.203, 0.327
	Unusual eating out	−0.001	−0.011, 0.010	0.092	−0.060, 0.258	−0.001	−0.331, 0.397

*ACME* average causal mediation effects, *ADE* average direct effects, *95* *% CI* 95 % confidence interval

## Discussion

In this study, we assessed whether the association of children’s socioeconomic status and dietary quality is mediated by their eating behaviours. We found that children with low socioeconomic status tended to have poorer dietary quality, but that this association was mediated by eating breakfast.

Dietary habits formed in childhood can persist into adulthood. For that reason, childhood is a critical period. Moreover, the dietary quality of children is directly related to their growth and development. There is no doubt that early-life interventions are effective, but the best way to improve children’s dietary quality remains questionable. To solve that problem, this study evaluated eating breakfast, family meals, and eating out as possible mediating factors, as suggested by other epidemiological studies [5, 8, 9].

Of these potential mediating factors, only eating breakfast showed a clear association with poor dietary quality, regardless of socioeconomic indicators, in this study. Similarly, Veugelers et al. [8] reported that skipping breakfast was associated with an approximately 18 % increase in the risk of a poor diet in children. On this basis, we assessed the mediation effect of eating breakfast on the association between socioeconomic indicators and children’s dietary quality. Its effect was significant when considering household income, but it only accounted for close to 7 % of the association. A recent Dutch study reported that parental intake of fruit and vegetables was a possible mediator of the relationship between children’s fruit and vegetable consumption and maternal education level [7]. A Norwegian study revealed that fruit and vegetable accessibility at home may account for the above relationship [16]. However, there was insufficient evidence to address whether dietary quality was mediated by their eating habits.

The other eating behaviours, eating breakfast and eating out, had significant associations with socioeconomic status. However, only eating breakfast was also associated with dietary quality; eating out was not. Socioeconomic factors, such as the price of food, income, education, time use, and grocery shopping trends, can influence food choices and dietary habits [17]. In general, childhood socioeconomic status is determined by parental education, parental occupation, and household income [18]. The 2010 study from European Childhood Obesity Surveillance Initiative reported that children with a high family income or parents with a higher educational level have a lower risk of consuming soft drinks with sugar and are more likely to eat breakfast and fresh fruit on a daily basis [19]. One multi-country study reported that children who did not eat breakfast on a daily basis were more likely to be from low-income households [20], but another study reported opposing results [21]. A study on the beneficial effects of breakfast reported that children who ate breakfast daily consumed more fruit and vegetables were more physically active, consumed fewer soft drinks, had lower risks of smoking and alcohol consumption, and spent less time watching TV [20]. Other evidence also suggests that eating breakfast is positively associated with scores regarding dietary variety [22]. Despite the several advantages of eating breakfast, a recent longitudinal study among 2- to 18-year-old children reported that the regular eating of breakfast has been gradually decreasing over time, from 84.8 % in 1986–1990 to 72.6 % in 2004–2007 [23]. Thus, people should be better informed about the benefits of eating breakfast and should encourage children to do so.

One study of the association between socioeconomic disparity and dietary quality reported that parental education level and occupational level were independently associated with dietary quality in adolescents from northern Europe [6]. Another study also reported that children or adolescents from relatively low-income households were less likely to consume the recommended amounts of micronutrients [5]. Several plausible explanations support a causal link between socioeconomic status and dietary quality. Paying for food or groceries depends on household incomes. Generally, a nutrient-dense diet is more expensive than an energy-dense diet [4]. Putnam et al. [24] reported that the cost of food accounted for 9 % of the income of higher-income households, whereas food accounted for 21–34 % of that of lower-income households. The association between parental educational and child dietary quality may be explained by the possibility that more highly educated parents have a better understanding of nutritional information and apply it more easily [6]. Another study found that mothers with high-level education were more likely to restrict their children’s intake of unhealthy foods [25]. Each socioeconomic indicator impacts dietary quality in different ways. Furthermore, education and occupation may have different meanings in different places and time periods [18, 26]. Household income represents a reliable socioeconomic indicator on a short-term basis. In this study, we used household income adjusted for family size to compare households. Our results also support the association between socioeconomic status and dietary quality, but there was no association between dietary quality and each parent’s educational level or occupation, even when examining employment status instead of occupation.

Our results reflect the design of the KNHANES survey; in fact, the chance of type I errors changes depending upon the survey design. To improve dietary quality in the service of promoting public health, we explored relevant modifiable factors using nationally representative survey data. However, this study had several limitations. We used 1-day dietary survey data to assess the dietary quality of each subject. This approach may have increased the possibility of misclassification bias, which would have resulted in an attenuated association. The use of a single 24-h recall period per individual is appropriate to characterize the average usual intake [27]. Although the quality of the KNHANES data was subjected to systematic review by expert committees and academic societies in Korea [10], the assessment of personal dietary intake via an interview regarding recalled intake during a single 24-h period involves issues of reliability and validity. Thus, it is necessary to have an understanding of the results. Nevertheless, our results showed significant effects of lower household income on children’s poor dietary quality as well as a mediation effect of eating breakfast. Our results were derived from a cross-sectional design study and therefore do not necessarily demonstrate a causal relationship.

## Conclusion

In conclusion, we found that part of children’s poor dietary quality was explained by eating behaviour associated with household income, but the direct effect of household income on children’s poor dietary quality was much greater than can be explained by associated eating habits alone. Our results may support the need for national interest in this issue and for funding to reduce income-related differences in the quality of the diet consumed by Korean children. Thus, economic vulnerability needs to be considered a priority in efforts to reduce the discrepancy in the dietary quality of children. Additionally, because causal mediation analysis revealed the positive effect of eating breakfast, we recommend that children eat breakfast.
